# Prevalence and Factors Associated With Syphilis in People Living With HIV/AIDS in the State of Pará, Northern Brazil

**DOI:** 10.3389/fpubh.2021.646663

**Published:** 2021-08-09

**Authors:** Pedro Leão Fontes Neto, Ricardo Roberto de Souza Fonseca, Maria Eduarda de Sousa Avelino, Elizandro Monteiro Vilhena, Maria dos Anjos de Abreu Pina Barbosa, Carmen Andrea Freitas Lopes, Samara Tatielle Monteiro Gomes, Bianca Jorge Sequeira, Rogério Valois Laurentino, Felipe Bonfim Freitas, Aldemir Branco Oliveira-Filho, Luiz Fernando Almeida Machado

**Affiliations:** ^1^Biology of Infectious and Parasitic Agents Post-Graduate Program, Federal University of Pará, Belém, Brazil; ^2^Virology Laboratory, Institute of Biological Sciences, Federal University of Pará, Belem, Brazil; ^3^Reference Unit Specialized in Infectious and Parasitic Diseases, Belém, Brazil; ^4^Evandro Chagas Institute, Health Ministry of Brazil, Ananindeua, Brazil; ^5^Study and Research Group on Vulnerable Populations, Institute for Coastal Studies, Federal University of Pará, Bragança, Brazil

**Keywords:** epidemiology, syphilis, HIV/AIDS, vulnerability, Amazon region

## Abstract

Syphilis continues to be a public health problem worldwide and its incidence has increased in people living with HIV/AIDS in recent years. This study determined the prevalence and factors associated with syphilis in people living with HIV/AIDS in the city of Belém, northern Brazil. A cross-sectional study was conducted from June to November 2018. A total of 500 people living with HIV/AIDS attended at a specialized unit of the public health network of the State of Pará were studied. Questionnaires were used to collect socio-demographic data and potential risk factors for syphilis. Blood samples were collected from all subjects and screened for syphilis using VDRL, and the seropositive were confirmed using FTA-abs. Logistic regressions were used to identify the factors associated with syphilis. Most subjects were male (56.8%), had more than 40 years (54.0%), single (63.0%), had finished high school (54.2%), had monthly income ≤1 minimum wage (72.4%), and had been born to the city of Belém (59.8%). Prevalence of syphilis was 6.4%. Eight characteristics/behaviors associated with syphilis: male, young adults, single, studied at least high school, monthly income >1 minimum wage, homosexual/bisexual, does not use or sporadically use condoms during sexual intercourse, and have had more than one sexual partner in the last three months. The prevalence of syphilis in people living with HIV/AIDS in Belém is low when compared to other Brazilian states. However, there is a need for public policies and actions to monitor, control and prevent these two sexually transmitted infections.

## Introduction

The Joint United Nations Program on HIV/AIDS (UNAIDS) estimates that approximately 2.1 million people are living with human immunodeficiency virus (HIV) in Latin America (1) and 1,011,617 acquired immunodeficiency syndrome (AIDS) cases were diagnosed in Brazil from 1980 to June 2020 (2). Northern Brazil has the second lowest reported HIV infection rate, with 30,943 (9.0%) cases reported between 2007 and June 2020 and maintains a linear trend of growth in the AIDS detection rate, which increased from 20.9 cases in 2009 to 26.0 cases/100 thousand inhabitants in 2019 (2). In this period, the Brazilian state of Pará (8,084 million inhabitants) had the highest cumulative number of cases (7,975 cases) of AIDS (2). Although the efficacy of highly active antiretroviral therapy (HAART) plays an important role in increasing the survival of people living with HIV/AIDS (PLWHA), the occurrence of co-infections such as syphilis is still a major concern worldwide (3).

Syphilis is caused by the bacterium *Treponema pallidum* being considered a serious public health problem. Globally, there are about 18 million people infected with *T. pallidum*, and approximately six million new cases are reported per year in people aged 15–49 (4). *T. pallidum* is transmitted primarily through unprotected sex. Being that, this bacterium can also be transmitted from the mother-child and through inadequate blood transfusion (5). In Brazil, 783,544 cases of syphilis were reported from 2010 to June 2020. In 2019, over 150,000 new cases of acquired syphilis have been diagnosed, approximately 61,000 cases in pregnant women and 24,000 cases of congenital syphilis. In the same period, almost 19,000 individuals with syphilis were diagnosed in northern Brazil, the second smallest number of records among Brazilian regions (6).

Some studies have shown an increase in the incidence of syphilis among young HIV-infected individuals in distinct regions of the world, reflecting the low adherence to condom use as a form of protection against sexually transmitted infections (STIs) (7, 8). This fact is very worrying because HIV can influence the course of infection by *T. pallidum*, resulting in a more aggressive disease and a worse treatment response (9). In Brazil, the prevalence of syphilis in PLWHA varies from region to region, being one of the most frequent coinfections in this group of vulnerable individuals. In Pernambuco (northeastern Brazil), the prevalence of coinfection was 8.8% (10), while in Rio de Janeiro (southeastern Brazil) it was 2.7% (11). In northern Brazil, little is known about the prevalence of syphilis in PLWHA.

Isolated data indicate that in 2018 the state of Pará had an acquired syphilis detection rate of 30.0 per 100,000 inhabitants (6), while the HIV detection rate was 19.0 (2). That is, high rates when compared to other states in Brazil. Thus, we established the prevalence of HIV/AIDS-syphilis co-infection in the city of Belém, state of Pará, as well as identifying the factors associated with syphilis, in order to contribute to the proposal of measures for the prevention and control of the two diseases in this Brazilian area and in others with socioeconomic and technological similarities.

## Materials and Methods

### Type of Study and Ethical Aspects

The present study is descriptive, cross-sectional and observational. The study was approved by the Research Ethics Committee with Human Beings of the Institute of Health Sciences of the Federal University of Pará, Belém, Brazil (Protocol number 2.601.161). Written informed consent was obtained from all PLWHA for the publication of any images or potentially identifiable data included in this article.

### Study Design

**Figure 1:**
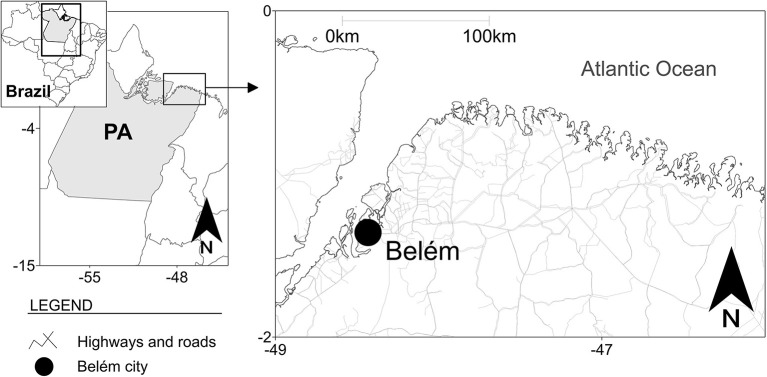
Geographic location of the city of Belém, state of Pará (PA), northern Brazil.

The study group included 500 PLWHA attending in the Specialized Reference Unit on Special Infectious and Parasitic Diseases (Unidade de Referência Especializada em Doenças Infecciosas e Parasitárias Especiais-UREDIPE) located in the city of Belém, capital of the state of Pará, northern Brazil (Figure 1) from June to November 2018. The Specialized Reference Unit is a State Reference Center for HIV/AIDS in Belém and is a testing and counseling center for carrying out tests for HIV, syphilis and other STIs. Performs clinical and laboratory monitoring of PLWHA, attending patients from most of the 144 municipalities of state of Pará.

The inclusion criteria were: ≥18 years old, confirmed HIV infection, all residents in the Pará State, who reported not having previous or current knowledge of having syphilis (confirmed in the medical record), who signed a free informed consent form and answered the epidemiological questionnaire. The exclusion criteria were patients with cognitive impairment who were unable to answer the questionnaire in an appropriate way, individuals who reported having had syphilis (either by self-report or by proof in medical records) and individuals who had active syphilis (confirmed by the laboratorial exams contained in the medical record).

The recruitment of study participants at UREDIPE was carried out on the day of the week when the PLWHA performed blood collection for the HIV-1 plasma viral load and quantification of CD4+/CD8+ T lymphocytes exams. During this waiting time, PLWHA were informed of the research through a brief lecture, in which the objectives of the study were clarified and invited to participate in it. The subjects who agreed to participate in the research signed a consent form. Social, demographic and behavioral information (age, sex, marital status, place of residence, schooling, monthly income, condom use during sexual practice, number of partners, sexual intercourse with sex worker, and history of STIs) were obtained through a semi-structured questionnaire. The rate of refusal to participate in the study was around 12.3%.

### Sample Size

The sample size determination was based on the estimated prevalence of syphilis in the general population and on the estimative of individuals enrolled in Specialized Reference Unit on Special Infectious and Parasitic Diseases in 2018 (approximately 7,420 patients, of which 5,219 were PLWHA). An estimated prevalence of 10% of active syphilis in PLWHA (12–14) resulted in a minimum sample size of 491 individuals. The sample error (ε) assumed in the present calculation was 5%, and a test power of 80% was established.

### Laboratory Tests

From each subject, a peripheral blood sample (5 mL) was collected by a vacuum collection system in a tube containing EDTA as anticoagulant. Plasma was separated by centrifugation (8,000 rpm for 15 min) and stored at −20°C until the moment of use, in the Laboratory of Virology, Institute of Biological Sciences, Federal University of Pará, where all laboratory tests were performed. Thereafter, each sample was submitted to the qualitative test for detecting antibodies (reagins) using Venereal disease research laboratory (VDRL, Wama Diagnostic Kit, Brazil), and dilution 1:8 to avoid the pro-zone effect. Samples with titers ≥1:8 using VDRL were evaluated by the Fluorescent treponemal antibody absorption (FTA-abs, Wama Diagnostic Kit, Brazil) to confirm the diagnosis. All subjects who had titers in VDRL ≥1:8 and were positive in FTA-abs had a confirmed diagnosis for syphilis, corroborating with the guidelines of the Ministry of Health, Brazil, for the diagnosis of syphilis (15).

### Statistical Analysis

All study data collected were entered into an Excel database, which was used for all statistical procedures and analysis. 95% confidence interval (95% CI) was determined to estimate the prevalence of syphilis. A descriptive analysis was conducted to investigate the bivariate relationships between syphilis and epidemiological (i.e., social, demographic or behavioral characteristics) covariates drawn from the epidemiological questionnaire data. All the potential epidemiological variables with probabilities of *p* ≤ 0.2 were examined and included in the model of factors associated to syphilis using backward stepwise multiple logistic regression (multivariate analysis). Various possible types of interactions were evaluated in order to determine how they might improve the final model. The fit of the final model was assessed using the Hosmer–Lemeshow goodness-of-fit test. A *p* < 0.05 significance value was considered for all analyses. All statistical analyses were performed using BioEstat 5.0 for Windows.

## Results

The average age was 39.3 years. According to age, PLWHA were divided into three classes: 18–30 years (*n* = 95, 19.0%), 31–40 years (*n* = 135, 27.0%), and over 40 years (*n* = 270, 54.0%). Most PLWHA were male (56.8%), had more than 40 years (54.0%), single (63.0%), had finished high school (54.2%), had monthly income of up to 1 minimum wage (72.4%), and had been born to the city of Belém (59.8%). In addition, most participants reported to be heterosexual (69.2%), but some individuals reported being homosexual (24.6%) and bisexual (6.2%). Many participants related having always used a condom during sexual intercourse in the last 3 months (61.6%). However, some of them still reported never having used (12.0%) or sporadically used condoms during sexual intercourse (26.4%). Most participants reported having had only one sexual partner in the last three months, reported not having had sex with sex workers and also reported having no history of STIs. The epidemiological information on the characteristics or behaviors of PLWHA is shown in Table 1.

**Table 1:** Epidemiological and behavioral characteristics of people living with HIV/AIDS, according to the diagnosis of syphilis, attended in the city of Belém, Pará, from June to November 2018.

**Characteristics/behaviors**	***N***	**Syphilis–(%)**	**Syphilis ^+^ (%)**	***p-value***
Total	500	468	32	-
Sex				
Male	284	255 (89.8)	29 (10.2)	<0.01^a^
Female	216	213 (98.6)	3 (1.4)	
Age group (years)				
18–30	95	83 ()	12 ()	<0.01^b^
31–40	135	125 ()	10 ()	
>40	270	260 ()	10 ()	
Civil Status^+^				
Single	315	288	27	0.03^c^
Married	144	140	4	
Divorced/Widowed	41	40	1	
Education				
Elementary school	159	155	4	<0.01^c^
High school	271	254	17	
University	70	59	11	
Family income^+^				
More than 1 minimum wage	138	123	15	0.02^b^
Up to 1 minimum wage^§^	362	345	17	
Source				
City of Belém	299	275	24	0.10^b^
Another city in Pará	201	193	8	
Sexual orientation				
Homosexual	123	103	20	<0.01^b^
Heterosexual	346	340	6	
Bisexual	32	25	6	
Use of condoms^+^				
Always	308	291	17	<0.01^c^
Never	60	60	0	
Sometimes	132	117	15	
Sexual partner number^+^				
None	124	118	6	0.03^b^
One	302	286	16	
> 1	74	64	10	
Sexual intercourse with sex worker^+^				
Yes	76	70	6	0.75^b^
No	424	398	26	
STIs History^+^				
Yes	207	194	13	0.93^a^
No	293	274	19	

+ *Last 3 months*.

§ *Around 210 US dollars/month;*

a *Fisher's exact test;*

b *Chi-square test;*

c *G test. STIs, Sexually Transmitted Infections*.

In total, 32 (6.4%; 95% CI = 1.8–11.0%) had a confirmed diagnosis of syphilis using VDRL and FTA-ABS. where a VDRL title greater than or equal to 1:8 with positive FTA-ABS was considered. Serologic testing showed that the VDRL titers was less than or equal to 1: 8 in 67.2% of individuals, between 1:16 and 1:64 in 23.9%, and ≥1:128 in 8.9%. Only one participant had a reaction with 1:512 (Supplementary Table 1). In addition, eight characteristics/behaviors were found to have an association with syphilis, based on bivariate and multivariate analyzes: male, up to 30 years, single, studied at least high school, monthly income higher than one minimum wage, homosexual or bisexual, does not use or sporadically use condoms during sexual intercourse, and have had more than one sexual partner in the last 3 months (Table 2). The Hosmer-Lemeshow goodness-of-fit test showed that the final model was a good fit (_HL_χ^2^ = 5.4; *p* = 0.6). Age up to 30 years (young adult) was identified as the principal risk factor for syphilis in PLWHA in the city of Belém (aOR = 28.3).

**Table 2:** Characteristics and behaviors of people living with HIV/AIDS associated with syphilis in the Brazilian city of Belém, Pará, northern Brazil, using bivariate and multivariate analysis.

**Characteristics/behaviors**	**N**	**Syphilis + (%)**	**Bivariate**	**Multivariate**
			**OR (95% CI)**	**aOR (95% CI)**
Male vs. Female	284	29 (10.2)	8.1 (2.3–25.6)	7.4 (1.9–18.5)
Up to 30 years *vs*. > 30 years	95	12 (12.6)	37.1 (19.5–87.8)	28.3 (14.2–63.8)
Not married *vs*. Married	356	28 (7.9)	4.0 (1.3–12.8)	3.7 (1.2 - 10.6)
High School + University *vs*. Elementary School	341	28 (8.2)	3.3 (1.2 - 9.9)	4.0 (1.3 - 11.3)
More than one minimum wage *vs*. Up to one minimum wage ^§^^+^	138	15 (10.9)	2.5 (1.1–5.0)	2.1 (1.2–6.7)
Homosexual + Bisexual *vs*. Heterosexual	154	26 (16.0)	11.4 (4.5–27.2)	12.1 (3.9–26.8)
Never + Sometimes *vs*. Always used a condom^+^	192	19 (9.9)	2.5 (1.2–5.2)	3.4 (1.3–5.0)
More than a sexual partner *vs*. Up to 1 (none included) ^+^	85	10 (11.8)	2.4 (1.1–5.3)	2.8 (1.1–4.8)

§ *Around 210 US dollars/month*.

+ *Last 3 months*.

*OR, Odds Ratio; aOR, adjusted Odds Ratio; 95% CI, 95% Confidence intervals*.

Among the epidemiological characteristics that have not been shown to be associated with the increased risk of acquiring syphilis are the patient's origin (whether from the capital or the interior of the state) and the history of STIs. Characteristics or behaviors not associated with syphilis in PLWHA in the city of Belém can be seen in the additional file (Supplementary Table 2).

## Discussion

The prevalence of syphilis detected in this study (6.4%) was higher than that found in PLWHA in Brazilian cities of Rio de Janeiro (2.7%) (11) and Vitória (5.3%) (12). However, higher prevalence of syphilis has also been detected in PLWHA in cities in the Brazilian states of Pernambuco (8.8%) (10), Paraná (15.9%) (3), Sergipe (9.1%) (14) and Rio Grande do Sul (20.5%) (16), and other countries, such as Germany (20.3%) (17) and Israel (15.2%) (18). Several factors (study design, sampling, laboratory tests and potentially temporal/local factors) may be interfering with these differences in the prevalence of syphilis. A comparison restricted to the city of Belém (Brazilian state of Pará) indicates that the prevalence of syphilis detected in this study is lower than the records made in two studies: newly diagnosed HIV therapy patients (17.3%) (19), and HIV/AIDS patients receiving care at the URE-DIPE from 2007 to 2008 (7.3%) (20). On the other hand, the number of PLWHA with higher titrations (1:128; 1:256; 1:512) using VDRL is higher in this study compared to that observed in the years 2007 and 2008 (20). Despite this reduction in the prevalence of syphilis in PLWHA, there is a clear need for planning and executing more efficient actions for the diagnosis, prevention and control of these two STIs.

This study affirms that sexual risk behavior and sociodemographic characteristics can facilitate the acquisition and spread of *T. pallidum* in PLWHA and possibly other population groups. Age up to 30 years (young adults) was identified as the main risk factor for syphilis in PLWHA in the city of Belém, despite the predominance of people over 40 years in the sample. This fact had not yet been observed in studies conducted in Brazil (11, 19, 20). It is an alert to the health authorities of the state of Pará and northern Brazil, because it is an indication that the methods of prevention of STIs are not being used adequately in the age group of beginning and, likely, of greater sexual activity. Ignorance or neglect of appropriate use of STIs preventive methods should be targets of public policies and health promotion actions.

Interestingly, most PLWHA involved in the study, as well as most co-infected individuals, had completed high school (>8 years of study). This shows a change in the profile of the target population, when compared to older studies, where the schooling reported by subjects was much lower (3, 4). An interesting association, indicated by the multivariate analysis, was the highest prevalence of co-infections among single and more than one sexual partner in the last 3 months, likely due to an association with age (<30 years) of the subjects. This demonstrates the lack of health care, especially with regard to prevention of STIs, and reinforces the need for interventions to promote health. The association of civil status (single) with syphilis in PLWHA has been reported in Brazil (21). Vulnerability to unplanned pregnancies and STIs, especially HIV and *T. pallidum* agents, has increased among adolescents and young adults in Brazil, even among those with only one sexual partner in the last 3 months, reflecting unprotected sexual practice (22), as well as registered here.

Effective interventions should be employed to promote sexual health by increasing the use of condoms. The effects of interventions to promote sexual health in young people tend to increase in the long-term, especially on behavioral and biological measures (23). In this sense, the theories of reasoned action and planned behavior have been shown to be more efficient, with self-efficacy models, with information motivation and behavioral skills; whereas interventions designed to induce fear do not (24). Specialized professionals from health institutions in the city of Belém, such as UREDIPE, should actively participate in the planning and execution of these interventions, including as facilitators, since when populations are not empowered, specialized facilitators are particularly effective, and are more effective if they also share the gender and ethnicity of the target audience.

The study has limitations and should be considered for interpretation of the results. One limiting factor was the restriction of the study to the city of Belém, which, although sharing many characteristics of the state of Pará and northern Brazil, small variations may not have been represented. The small sample size may not necessarily be representative of the actual rate of HIV/Syphilis co-infection in the state of Pará, Brazil. In addition, although convenience sampling was considered adequate for sampling that was almost representative of the investigated population, other sampling methods should be applied to improve representativeness. As interview data are self-reported, some information, such as drug use or sex-related risks behaviors, may contain response or recall bias, especially associated with shame or fear of discrimination. Finally, the cross-sectional design of the study limits its capacity to establish causality.

In conclusion, this study detected a low prevalence of syphilis in PLWHA in the city of Belém, Pará, northern Brazil. The relevant characteristics of the co-infected by HIV and *T. pallidum* were established and should be used for the control and prevention of infections caused by these pathogens, especially by local authorities to promote the health of the general population and PLWHA.

## Data Availability Statement

The original contributions presented in the study are included in the article/Supplementary Material, further inquiries can be directed to the corresponding author.

## Ethics Statement

The studies involving human participants were reviewed and approved by Research Ethics Committee with Human Beings of the Institute of Health Sciences of the Federal University of Pará, Belém, Brazil (Protocol number 2.601.161). The patients/participants provided their written informed consent to participate in this study.

## Author Contributions

PN and LM: conceptualization. PN, MA, RF, EV, MB, and CL: data curation. PN, MA, RF, MB, EV, SG, and FF: investigation and methodology. BS, RL, and AO-F: formal analysis. PN, RL, and LM: writing—original draft. AO-F and LM: writing—review and editing. LM: project administration. All authors contributed to the development of research, read, and approved the final manuscript.

## Conflict of Interest

The authors declare that the research was conducted in the absence of any commercial or financial relationships that could be construed as a potential conflict of interest.

## Publisher's Note

All claims expressed in this article are solely those of the authors and do not necessarily represent those of their affiliated organizations, or those of the publisher, the editors and the reviewers. Any product that may be evaluated in this article, or claim that may be made by its manufacturer, is not guaranteed or endorsed by the publisher.
