# Supplementary material for: Prevalence and Factors Associated With Syphilis in People Living With HIV/AIDS in the State of Pará, Northern Brazil
Source: Front Public Health. 2021 Aug 9;9:646663. doi: 10.3389/fpubh.2021.646663 (PMC8381150; doi:10.3389/fpubh.2021.646663)
Supplement: Supplementary file 1 [file Table_1.docx]

**Table S1.** VDRL results and titers in people living with HIV/AIDS in the city of Belém, Pará, northern Brazil.

| **VDRL** | **N (%)** |
| --- | --- |
| Plasma (n = 500) |  |
| Positive | 67 (13.4) |
| Negative | 433 (86.6) |
| Titers (n = 67) |  |
| 1:1 | 14 (20.9) |
| 1:2 | 15 (22.4) |
| 1:4 | 5 (7.4) |
| 1:8 | 11 (16.4) |
| 1:16 | 8 (11.9) |
| 1:32 | 4 (6.0) |
| 1:64 | 4 (6.0) |
| 1:128 | 4 (6.0) |
| 1:256 | 1 (1.5) |
| 1:512 | 1 (1.5) |
