# Supplementary material for: Prevalence and Factors Associated With Syphilis in People Living With HIV/AIDS in the State of Pará, Northern Brazil
Source: Front Public Health. 2021 Aug 9;9:646663. doi: 10.3389/fpubh.2021.646663 (PMC8381150; doi:10.3389/fpubh.2021.646663)
Supplement: Supplementary file 2 [file Table_2.docx]

**Table S2**. Characteristics or behaviors of people living with HIV/AIDS not associated with syphilis in the city of Belém, Pará, northern Brazil, using bivariate and multivariate analysis.

| Characteristics/behaviors | N | Syphilis + (%) | Bivariate | Multivariate |
| --- | --- | --- | --- | --- |
|  |  |  | OR (95% CI) | cOR (95% CI) |
| City of Belém *versus* (vs.) Another city in Pará | 299 | 24 (8.0) | 2.1 (0.9 - 4.8) | 1.8 (0.8 - 4.0) |
| Had sexual intercourse with sex worker ^+^ *vs.* Did not have | 76 | 6 (7.8) | 1.3 (0.5 - 3.2) | 1.6 (0.7 - 3.6) |
| STIs History^+^ *vs.* No history | 207 | 13 (6.3) | 0.9 (0.5 - 2.00) | 1.1 (0.6 - 2.3) |

^+^ Last 3 months. OR: Odds Ratio. cOR: crude OR. 95% CI: 95% confidence interval.
